# Erstdiagnose ALCAPA-Syndrom im Erwachsenenalter – seltene Ursache eines Herz-Kreislauf-Stillstands

**DOI:** 10.1007/s00108-024-01782-4

**Published:** 2024-09-20

**Authors:** Tim Urban, Sebastian Grundmann, Franziska Klein, Tobias Wengenmayer, Katharina Müller-Peltzer, Hans-Jörg Busch

**Affiliations:** 1 Zentrum für Notfall- und Rettungsmedizin, Universitätsklinikum Freiburg, Sir-Hans-A.-Krebs-Straße, 79106 Freiburg, Deutschland; 2 Klinik für Kardiologie und Angiologie, Universitäts-Herzzentrum Freiburg – Bad Krozingen, Freiburg, Deutschland; 3 Interdisziplinäre Medizinische Intensivtherapie, Universitätsklinikum Freiburg, Freiburg, Deutschland; 4 Klinik für Diagnostische und Interventionelle Radiologie, Universitätsklinikum Freiburg, Freiburg, Deutschland

**Keywords:** Koronare Fehlbildung, Bland-White-Garland-Syndrom, Koronaranomalie, Reanimation, Plötzlicher Herztod, Resuscitation room management, Bland-White-Garland syndrome, Coronary anomalies, Cardiopulmonary resuscitation, Sudden cardiac death

## Abstract

Ein 42-jähriger Patient wurde uns notärztlich in den Schockraum zugewiesen nach „return of spontaneous circulation“ (ROSC) bei außerklinischem Herz-Kreislauf-Stillstand mit Kammerflimmern als initialem Rhythmus. Als Ursache präsentierte sich in der CT-Bildgebung (Computertomographie) und Koronarangiographie die Erstdiagnose eines ALCAPA-Syndroms („anomalus left coronary artery origin from pulonary artery“). Es zeigte sich eine typische kaliberstarke rechtskoronare Versorgung des Herzens und ausgeprägte Kollateralisierung. Nach initialer Intensivtherapie erfolgte die operative Versorgung des Befunds. Der Patient konnte ohne neurologisches Defizit in gutem Allgemeinzustand entlassen werden.

**Video online:**

Die Online-Version dieses Beitrags (10.1007/s00108-024-01782-4) enthält ein Video.

## Abstract (en)

A 42-year-old patient with return of spontaneous circulation (ROSC) following an out-of-hospital cardiac arrest was referred to the authors’ emergency department. The initial rhythm was ventricular fibrillation. A computed tomography scan and subsequent coronary angiography revealed anomalous left coronary artery from the pulmonary artery (ALCAPA) syndrome as the cause of this condition. A thickened right coronary artery with significant collateral blood flow to the left coronary artery was observed. After initial treatment in the authors’ intensive care unit, surgical intervention was performed. The patient was discharged from hospital without any neurological damage.

## Hintergrund

**Abb. 1:**
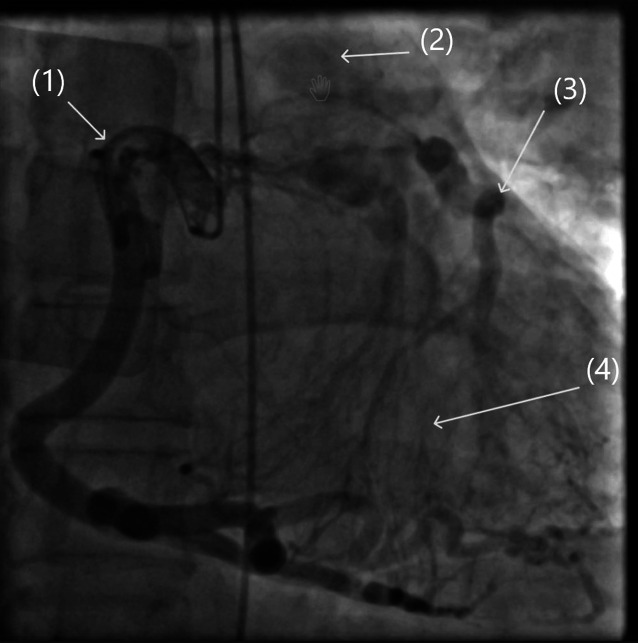
Koronarangiographie mit kaliberstarker rechter Koronararterie (*1*), ausgeprägter Kollateralisierung (*4*), sich retrograd füllender LAD (*3*) und Kontrastmittelabfluss im Truncus pulmonalis (*2*)

Das ALCAPA-Syndrom („anomalous left coronary artery origin from pulmonary artery“) ist eine angeborene Koronaranomalie, bei der die linke Koronararterie aus dem Truncus pulmonalis entspringt. Mit einer Inzidenz von 1:300.000 gehört sie zu den seltenen angeborenen Koronaranomalien. Meist fallen bereits im Kindesalter eine eingeschränkte Belastbarkeit und thorakale Schmerzsymptome aufgrund der unzureichenden myokardialen Perfusion mit sauerstoffreichem Blut auf [1, 2] Seltener fallen betroffene Patienten erst im Erwachsenenalter auf. Diese weisen oft eine ausgeprägte Kollateralisierung über das rechtskoronare Gefäßsystem auf. Aufgrund des Blutabflusses in das pulmonale Niederdrucksystem trägt nur ein Teil des kollateralen Blutflusses zur myokardialen Perfusion bei (Abb. 1; [3, 4]). Erwachsene entwickeln meist im frühen Erwachsenenalter eine Herzinsuffizienz [2]. Wir stellen einen Fall vor, bei dem die Diagnose erst mit 42 Jahren im Rahmen der Schockraumversorgung nach Reanimation bei außerklinischem Herz-Kreislauf-Stillstand gestellt wurde.

## Anamnese

Ein 42-jähriger Mann wurde durch den Notarzt als Schockraumpatient angemeldet. Der Mann sei plötzlich im Bad zusammengebrochen. Sein Sohn, der den Vorfall beobachtete, habe sofort den Rettungsdienst verständigt. Es erfolgten keine Basic-life-support(BLS)-Maßnahmen. Der Rettungsdienst traf nach 5 min ein und führte für weitere 6 min Advanced-life-support(ALS)-Maßnahmen durch. Der initiale Rhythmus war ein Kammerflimmern, das nach der dritten Defibrillation beendet werden konnte. Bei Glasgow Coma Scale (GCS) 6 erfolgte die Intubation vor Ort durch den Notarzt. Im Notarzt-EKG zeigte sich kein STEMI oder STEMI-Äquivalent. Prodromi oder Symptomatik vor dem Kammerflimmern wurden nicht berichtet. Der Patient war nicht vorerkrankt. Aufgrund der unklaren Ursache des Kammerflimmerns entschieden wir uns für eine primäre Versorgung in unserem interdisziplinären Schockraum mit „cardiac arrest receiving team“ (CART; [5]).

## Befunde

Bei Aufnahme im Schockraum präsentierte sich der Patient mit gesichertem Atemweg. Es zeigte sich eine gute Oxygenierung mit normalem CO_2_ unter Spontan-CPAP/ASB mit geringem Unterstützungsdruck. Der Patient zeigte normotensive Blutdruckwerte und einen normofrequenten Sinusrhythmus. Echokardiographisch zeigten sich keine Anzeichen für Rechtsherzbelastung, eine normale LV-Funktion sowie keine Klappenfehler und kein Perikarderguss. Die Pupillen waren beidseits eng und lichtreagibel, die FAST-Sonographie („focused assessment with sonography for trauma“) blieb ohne Hinweis auf Trauma oder Reanimationsursache. In der BGA zeigte sich kein Hinweis auf eine Reanimationsursache.

## Diagnose

**Abb. 2:**
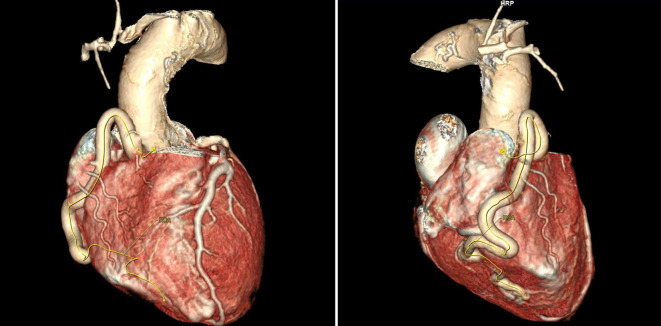
Zwei Ansichten der 3‑D-Rekonstruktion des Kardio-CT mit pathognomonischer kaliberstarker und mäandernder RCA

- Bei weiterhin unklarer Reanimationsursache entschieden wir uns für eine Computertomographie von Schädel/HWS/Abdomen und Thorax. Hier zeigte sich als Ursache des Kammerflimmerns ein bis dahin nicht diagnostiziertes ALCAPA-Syndrom (Abb. 2 und 3; [6]). In der im Verlauf durchgeführten Koronarangiographie zeigte sich der typische Befund eines sehr kaliberstarken und mäandernden rechtskoronaren Gefäßes mit ausgeprägter Kollateralisierung zur linken Koronararterie (Abb. 1; [7]).

**Abb. 3:**
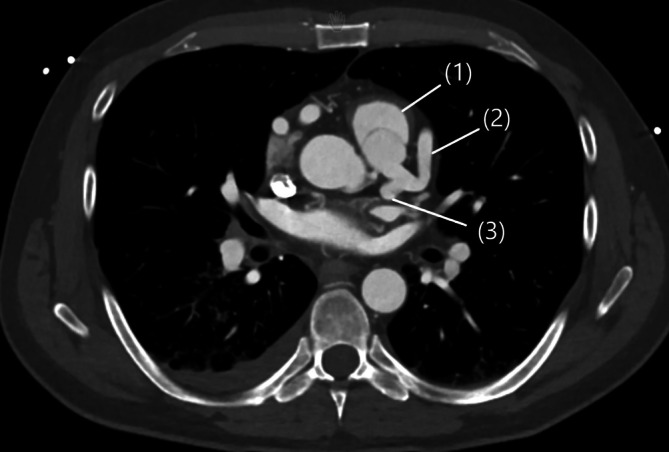
Truncus pulmonalis (*1*) mit Fehlmündung des linken Hauptstamms bestehend aus LAD (*2*) und LCX (*3*)

## Therapie und Verlauf

**Abb. 4:**
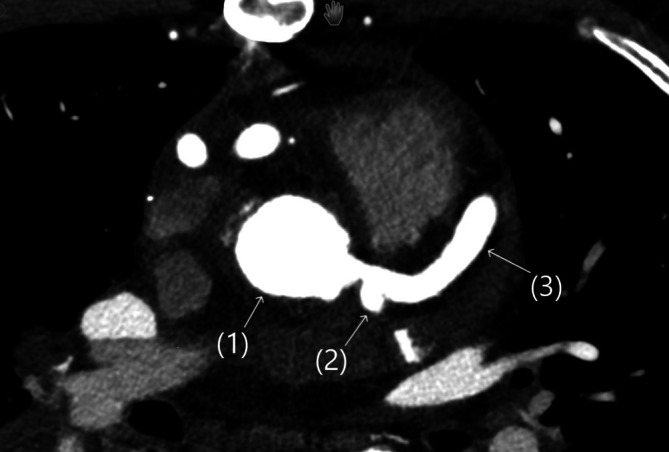
Aorta ascendens (*1*) nach Reimplantation von LCX (*2*) und LAD (*3*)

Nach standardisierter Postreanimationsbehandlung auf unserer Intensivstation konnte der Patient rasch extubiert werden. Wenige Tage später erfolgte der problemlose Koronartransfer des linken Koronarostiums vom LPA-Stamm zur Aortenwurzel (Abb. 4; [8]). Es erfolgte die sekundärprophylaktische Implantation eines ICD. Der Patient ist normal belastbar und lebt ohne neurologisches Defizit mit seiner Familie im Schwarzwald.

## Diskussion

Aufgrund der geringen Symptomatik im Vorfeld ist davon auszugehen, dass der Patient eine sehr gute Kollateralisierung über das rechtskoronare System gehabt haben muss. Es zeigten sich keine typischen EKG-Veränderungen wie ST-Strecken-Veränderungen der anterolateralen Ableitungen oder Q‑Zacken in den Ableitungen I, aVL, V5 und V6 [9]. Auch echokardiographisch wäre eine linksventrikuläre Dilatation mit konsekutiver Mitralklappeninsuffizienz zu erwarten gewesen [6]. Retrospektiv konnte erhoben werden, dass der Patient bereits in den Tagen vor der Reanimation immer wieder AP-Symptomatik und Übelkeit verspürt hatte. Im Wissen dieser Symptomatik wäre der Patienten nach Standard Operating Procedure im Herzkatheterlabor entgegengenommen worden [6]. Hier wäre das linke Koronarostium nicht darstellbar gewesen. Nach Gabe von ausreichend Kontrastmittel zeigte sich über die Kollateralisierung die retrograde Füllung der LAD mit Abfluss des Kontrastmittels ins Niederdrucksystem des Truncus pulmonalis (Koronarangiographie). Ein erfahrener interventioneller Kardiologe hätte auch mittels Koronarangiographie die Diagnose stellen können. Durch die CT-Bildgebung konnten in unserem Fall jedoch direkt die Weichen zur operativen Versorgung des Patienten gestellt werden. Beide Methoden haben ihren Stellenwert in der Diagnostik eines Herz-Kreislauf-Stillstands. Die weitaus häufigere Erkrankung, die zu einem Herz-Kreislauf-Stillstand führt, ist die koronare Herzkrankheit, hier ist die Koronarangiographie diagnostisch, aber vor allem therapeutisch einer Kardio-CT überlegen. Für unseren Patienten mit Koronaranomalie war die CT-Bildgebung jedoch die optimale Diagnostik. Für das sehr gute Outcome des Patienten war insbesondere auch die zeitnahe und suffiziente präklinische Versorgung maßgeblich [10].

## Fazit für die Praxis

- Seltene kardiale Anomalien als Reanimationsursache können in der CT-Bildgebung häufig besser als in der Koronarangiographie dargestellt werden.
- CPR-Patienten ohne eindeutige Hinweise auf STEMI oder STEMI-Äquivalent sollten von einem interdisziplinären Aufnahmeteam (CART) im Schockraum zur erweiterten Diagnostik entgegengenommen werden.
- Verschiedenste Ursachen können zu einem Herz-Kreislauf-Stillstand führen und müssen so rasch wie möglich diagnostiziert und behandelt werden. Ein schnelles Erkennen von Prioritäten und ein rasches, standardisiertes diagnostisches Vorgehen, insbesondere der vermutlich zum Ereignis führenden Erkrankung sowie reanimationsbedingter Verletzungen, stehen hierbei im Vordergrund.

## Video

Koronarangiografie mit Darstellung der starkkalibrigen RCA, ausgeprägter Kollateralisierung, retrogradem Fluss in der LAD und Kontrastmittelabfluss über den Truncus pulmonalis

## References

[1] Bland EF, White PD, Garland J (1933) Congenital anomalies of the coronary arteries: report of an unusual case associated with cardiac hypertrophy. Am Heart J 8:787

[2] Wesselhoeft H, Fawcett JS, Johnson AL (1968) Anomalous origin of the left coronary artery from the pulmonary trunk. Its clinical spectrum, pathology, and pathophysiology, based on a review of 140 cases with seven further cases. Circulation 38(2):403 5666852 10.1161/01.cir.38.2.403

[3] Edwards JE (1964) The direction of blood flow in coronary arteries arising from the pulmonary trunk. Circulation 29:163 14119382 10.1161/01.cir.29.2.163

[4] Wright NL, Baue AE, Baum S, Blakemore WS, Zinsser HF (1970) Coronary artery steal due to an anomalous left coronary artery originating from the pulmonary artery. J Thorac Cardiovasc Surg 59(4):461 5439356

[5] Hauw-Berlemont C, Lamhaut L, Diehl JL, Andreotti C, Varenne O, Leroux P, Lascarrou JB, Guerin P, Loeb T, Roupie E, Daubin C, Beygui F, Boissier F, Marjanovic N, Christiaens L, Vilfaillot A, Glippa S, Prat JD, Chatellier G, Cariou A, Spaulding C, EMERGE Investigators (2022) Emergency vs delayed coronary angiogram in survivors of out-of-hospital cardiac arrest: results of the randomized, multicentric EMERGE trial. JAMA Cardiol 7(7):700–707. 10.1001/jamacardio.2022.1416 35675081 10.1001/jamacardio.2022.1416 PMC9178496

[6] Srinivasan KG, Gaikwad A, Kannan BR, Ritesh K, Ushanandini KP (2008) Congenital coronary artery anomalies: diagnosis with 64 slice multidetector row computed tomography coronary angiography: a single-centre study. J Med Imaging Radiat Oncol 52(2):148 18373806 10.1111/j.1440-1673.2008.01933.x

[7] Pandey NN, Sinha M, Sharma A, Rajagopal R, Bhambri K, Kumar S (2019) Anomalies of coronary artery origin: Evaluation on multidetector CT angiography. Clin Imaging 57:87–98. 10.1016/j.clinimag.2019.05.010 31170598 10.1016/j.clinimag.2019.05.010

[8] Backer CL, Stout MJ, Zales VR, Muster AJ, Weigel TJ, Idriss FS, Mavroudis C (1992) Anomalous origin of the left coronary artery. A twenty-year review of surgical management. J Thorac Cardiovasc Surg 103(6):1049 1597969

[9] Hoffman JI (2013) Electrocardiogram of anomalous left coronary artery from the pulmonary artery in infants. Pediatr Cardiol 34(3):489–491 23242106 10.1007/s00246-012-0599-7

[10] Busch H‑J et al (2014) Der Herz-Kreislauf-Stillstand ist ein eigenständiges Krankheitsbild. Notfall Rettungsmed 17(4):281–286

